# Transfer of rice mitochondrial ribosomal protein L6 gene to the nucleus: acquisition of the 5'-untranslated region via a transposable element

**DOI:** 10.1186/1471-2148-8-314

**Published:** 2008-11-14

**Authors:** Nakao Kubo, Masaru Fujimoto, Shin-ichi Arimura, Masashi Hirai, Nobuhiro Tsutsumi

**Affiliations:** 1 Graduate School of Agriculture, Kyoto Prefectural University, Seika, Kyoto 619-0244, Japan; 2 Graduate School of Agricultural and Life Sciences, The University of Tokyo, Yayoi, Bunkyo-ku 113-8657, Japan

## Abstract

**Background:**

The mitochondria of contemporary organisms contain fewer genes than the ancestral bacteria are predicted to have contained. Because most of the mitochondrial proteins are encoded in the nucleus, the genes would have been transferred from the mitochondrion to the nucleus at some stage of evolution and they must have acquired cis-regulatory elements compatible with eukaryotic gene expression. However, most of such processes remain unknown.

**Results:**

The ribosomal protein L6 gene (*rpl6*) has been lost in presently-known angiosperm mitochondrial genomes. We found that each of the two rice *rpl6 *genes (*OsRpl6-1 *and *OsRpl6-2*) has an intron in an identical position within the 5'-untranslated region (UTR), which suggests a duplication of the *rpl6 *gene after its transfer to the nucleus. Each of the predicted RPL6 proteins lacks an N-terminal extension as a mitochondrial targeting signal. Transient assays using green fluorescent protein indicated that their mature N-terminal coding regions contain the mitochondrial targeting information. Reverse transcription-PCR analysis showed that *OsRpl6-2 *expresses considerably fewer transcripts than *OsRpl6-1*. This might be the result of differences in promoter regions because the 5'-noncoding regions of the two *rpl6 *genes differ at a point close to the center of the intron. There are several sequences homologous to the region around the 5'-UTR of *OsRpl6-1 *in the rice genome. These sequences have characteristics similar to those of the transposable elements (TE) belonging to the *PIF*/Harbinger superfamily.

**Conclusion:**

The above evidences suggest a novel mechanism in which the 5'-UTR of the transferred mitochondrial gene was acquired via a TE. Since the 5'-UTRs and introns within the 5'-UTRs often contain transcriptional and posttranscriptional cis-elements, the transferred rice mitochondrial *rpl6 *gene may have acquired its cis-element from a TE.

## Background

Mitochondria are thought to be descendants of endosymbiotic bacteria that entered into the host cell [1]. The mitochondria of contemporary organisms contain considerably fewer genes than the ancestral bacteria are predicted to have contained. Thousand or more mitochondrial proteins are predicted to be encoded in the nucleus [2,3]. Such the nucleus-encoded genes are transcribed from eukaryotic promoters, followed by translation into proteins by cytosolic ribosomes. In many cases, the proteins are synthesized as precursors having N-terminal extensions (presequences), which act as mitochondrial targeting signals. Most of these genes would have been transferred from the mitochondrion to the nucleus at some stage of evolution although some genes may have been recruited from other sources [4]. The transferred mitochondrial genes must have acquired cis-regulatory elements compatible with eukaryotic gene expression (e.g., promoters, enhancers, poly (A) signals and sequences for mitochondrial targeting signals) because mitochondrial gene expression is mainly prokaryotic. However, most of the processes for the gene activation remain unknown.

Mitochondrial gene content is highly variable depending on the taxa studied. The mammalian mitochondrial genome is conserved and constant all over the groups, whereas within Tracheophyta (higher plants), the genomes exhibit differential gene losses, indicating that gene transfer to the nucleus is an ongoing process during the evolution of Magnoliophyta (angiosperms) [5]. Typical such cases are the ribosomal protein genes, showing more frequent gene-loss than other types of mitochondrial gene in many angiosperm species. For example, a sequence homologous to the ribosomal protein L6 gene (*rpl6*) is absent from all known angiosperm mitochondrial genomes [6-8], whereas the corresponding sequence is encoded in the mitochondrial genomes of lower plants [9]. The sequences of the nucleus-encoded *rpl6 *gene have recently been identified in the complete *Arabidopsis *nuclear genome [6,8] and the draft rice nuclear genome [8]. However, detailed analysis has not yet been performed. We previously reported the loss or dysfunction of several ribosomal protein genes in the complete rice mitochondrial genome [10]. We have also isolated several genes that had been transferred from the mitochondrion to the nucleus in rice [11-14]. Previous studies, including ours, have revealed frequencies of gene transfer events, the origins of sequence elements, and a few possible mechanisms involved [5]. For examples, the *rps10 *gene has undergone numerous independent gene transfer events during recent angiosperm evolution [15]. Presequences for rice *rps11-1*, *Arabidopsis sdh3 *and carrot *rps10 *genes seem to have been copied from those for the *atp2, hsp70 *and *hsp22 *genes, respectively [11,15,16]. Common use of a presequence in different proteins via alternative splicing has also been found in maize and rice [12,17]. Chromosomal recombinations would have been involved in the gain of a promoter for rice *rpl27 *gene [18]. Genes are sometimes divided into pieces or functionally replaced: a coding region of *rpl2 *gene has been divided into 5'- and 3'-parts in dicots, either or both of which have been transferred to the nucleus in some species [19]; mitochondrial *rps13 *and *rps8 *genes have been replaced by duplicated copies of chloroplast (*rps13*) and cytosolic counterparts (*rps15A*), respectively [6]. However, despite these examples, it is mostly unclear how the sequence elements compatible with eukaryotic expression were successfully moved and then joined with the transferred mitochondrial genes.

In this study, we identified and characterized the rice *rpl6 *gene. The release of the complete nuclear sequence of rice [20] and its fine genome annotation [21] enabled us to survey the genes and their genomic environment in detail. Based on this information, two copies of rice *rpl6 *gene (*OsRpl6-1 *and *OsRpl6-2*) were identified in the rice genome. Sequence comparison of the two *rpl6 *genes strongly suggests a duplication of the *rpl6 *gene via genomic DNA rather than two separate gene transfer events. Although the sequences of the two *rpl6 *copies are homologous within the coding regions and have similar mitochondrial targeting properties, *OsRpl6-1 *was expressed to a greater extent than *OsRpl6-2*. A region around the 5'-untranslated region (UTR) of *OsRpl6-1 *is conserved in several other rice sequences. Interestingly, this conserved region has characteristics similar to those of class II transposable elements (TEs). It is well established that numerous TEs are present in eukaryotic nuclear genomes and that some of them affect genomic rearrangement and gene expression via translocation [22]. The TE within *OsRpl6-1 *would have been involved in the acquisition of the 5'-UTR, which may be responsible for the difference in the amount of transcripts produced by the two *rpl6 *genes. The significance of TEs for the activation of transferred mitochondrial sequence and the evolution of such processes are discussed.

## Results

### Identification of two copies of the mitochondrial rpl6 gene in the rice genome

**Figure 1:**
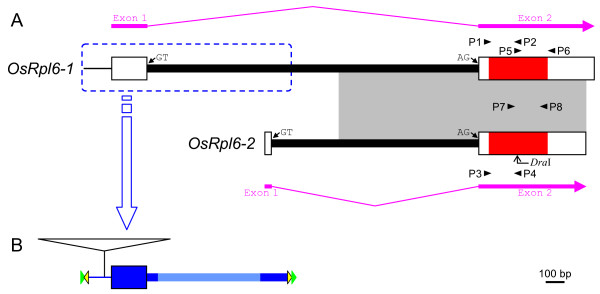
**Schematic representation of the rice mitochondrial *rpl6 *genes, *OsRpl6-1 and OsRpl6-2***. (A) The exon-intron structures of *OsRpl6-1 *and *OsRpl6-2*. Exons and introns are represented by boxes and thick lines, respectively. In the exons, the RPL6-protein coding regions are colored with red, whereas the 5'- and 3'-untranslated regions (UTRs) are colored with white. The 5'-nontranscribed spacer upstream of the *OsRpl6-1 *exon 1 is indicated by a thin line. The GT and AG dinucleotides at the border of the intron are indicated by small arrows. The direction of each *OsRpl6-1 *and *OsRpl6-2 *transcript is indicated by a thick pink arrow joined with a thin pink broken line. A homologous region in the two *rpl6 *genes is shaded. The locations of primers P1-P8 are indicated by small arrowheads. The position of the *Dra*I site in *OsRpl6-2 *is indicated by a bent arrow. The location of transposable elements (TEs) is enclosed with a blue dotted outline, and the structures are shown below. (B) Structure of TEs in *OsRpl6-1*. Blue and pale blue coloring, respectively, represent well-conserved and poorly-conserved regions among various rice sequences (Figs. 4 and 5). Potential terminal inverted repeat (TIR) and target site duplication (TSD) are indicated by yellow and green triangles, respectively (not to scale). An insertion of the *Mutator*-like element within the 5'-nontranscibed spacer region is indicated by an open triangle.

A BLAST search of the complete rice nuclear sequence [20] identified two *rpl6 *sequences, *OsRpl6-1 *and *OsRpl6-2 *(Fig. 1A). *OsRpl6-1 *is a newly described rice *rpl6 *gene, whereas the sequence of *OsRpl6-2 *corresponds to that of a previously reported one [8]. The *OsRpl6-1 *and *OsRpl6-2 *genes are assigned to chromosomes (Chrs) 3 and 8 as loci Os03g0725000 and Os08g0484301, respectively, in the Rice Annotation Project Database (RAP-DB) Build 4 [21]. Each of the *OsRpl6-1 *and *OsRpl6-2 *genes is transcribed because cDNA sequence corresponding to each gene is found in the database [representative GenBank accession nos. AK119694 and CI260120 for *OsRpl6-1 *and *OsRpl6-2*, respectively] (Fig. 1A, thick pink arrow joined with thin pink broken line). Each gene has an intron in an identical position within the 5'-UTR (Fig. 1A), as is the case for *Arabidopsis rpl6 *[8]. The coding regions of the two rice *rpl6 *genes, each of which is predicted to encode a protein consisting of 103 amino acids, have 92% nucleotide sequence identity. Because the 3'-terminal regions of the intron showed 65% identity in the two rice *rpl6 *(Fig. 1A, shaded region), they were probably generated by a duplication event via genomic DNA after gene transfer to the nucleus, rather than by two separate transfer events.

### Mitochondrial targeting of OsRpl6 gene products

**Figure 2:**
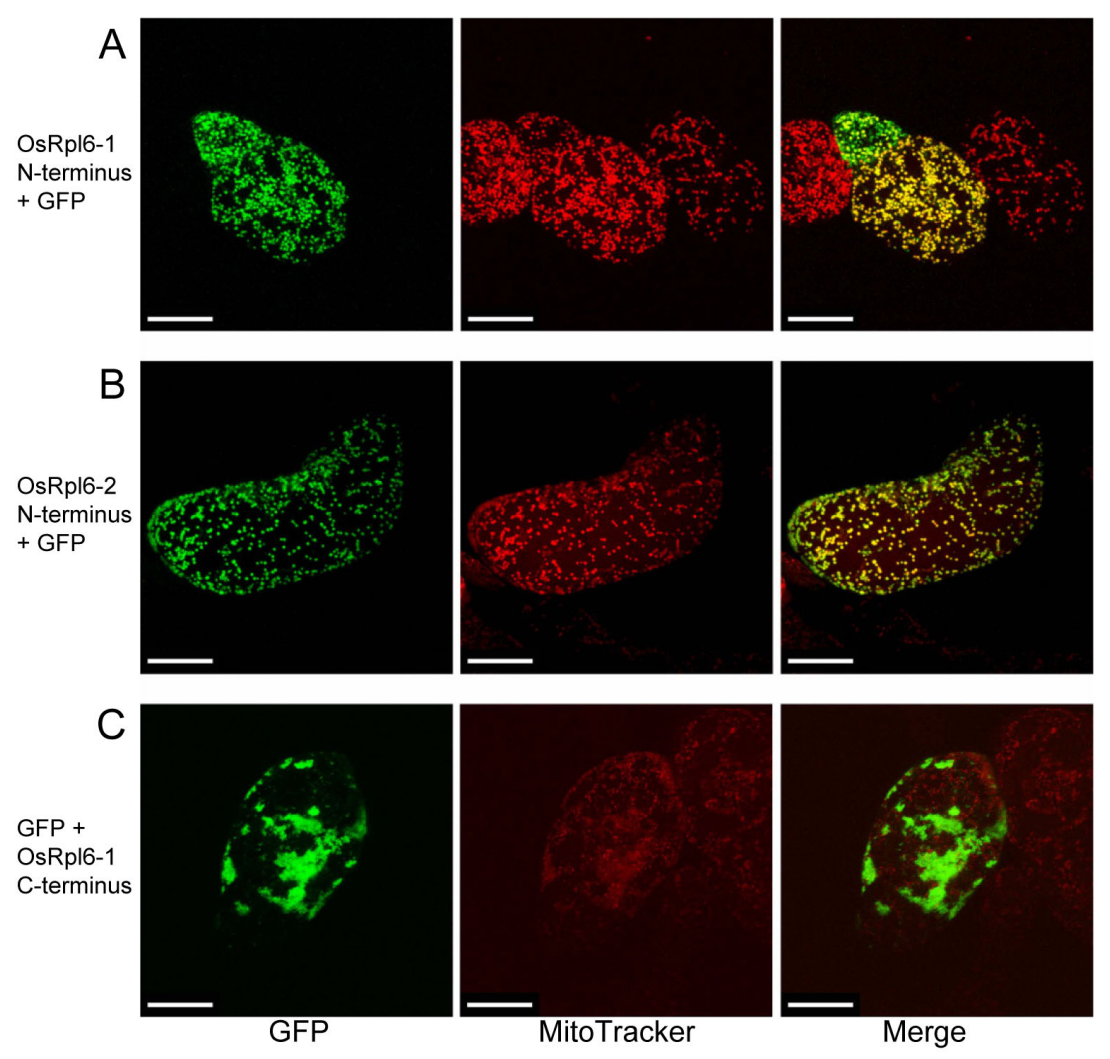
**Subcellular localization of GFP fusion proteins in tobacco BY-2 cells**. Constructs were fused to GFP cDNA at the following positions: (A) the N-terminal coding region of *OsRpl6-1 *was fused to 5'-upstream position of GFP, (B) the N-terminal coding region of *OsRpl6-2 *was fused to 5'-upstream position of GFP, and (C) the C-terminal coding region of *OsRpl6-1 *was fused to 3'-downstream position of GFP. Representative images are shown. Left: GFP fluorescence. Center: fluorescence of a mitochondrial-specific dye, MitoTracker Red. Right: merging of both signals. Scale bar = 20 μm. In the right panel of Figure 2A, some of the GFP fluorescence within a small cell behind the central cell did not co-localize with mitochondria. The GFP fluorescence from the behind cell would have been overexposed, probably because its GFP expression had been much more enhanced than that in the central cell.

In contrast to the mitochondrial RPL6 proteins of lower plants [9], the predicted proteins of the two rice *rpl6 *genes did not contain N-terminal extensions for presequences. Although the *Arabidopsis rpl6 *gene [8] and all the other Spermatophyta (seed plants) *rpl6 *cDNAs in the database also lack any coding capacity for presequences (data not shown), localization of the RPL6 protein has not been studied. We examined the subcellular localization of RPL6 using green fluorescent protein (GFP). A construct, in which the N-terminal coding region of *OsRpl6-1 *was fused to synthetic GFP cDNA [23], exhibited fluorescence in particles of about 1 μm in diameter (Fig. 2A, left panel). These particles coincided with the fluorescence of a mitochondrial-specific dye, MitoTracker Red (Fig. 2A, center and right panels), indicating that the protein was imported into the mitochondria. A construct containing the N-terminal coding region of *OsRpl6-2 *gave similar results (Fig. 2B). In contrast, a construct containing the C-terminal coding region of *OsRpl6-1 *did not localize proteins to the mitochondria (Fig. 2C). These results indicate that the mature N-terminal coding regions of rice RPL6 proteins are important for mitochondrial localization.

### Differential expression of rice rpl6 genes

**Figure 3:**
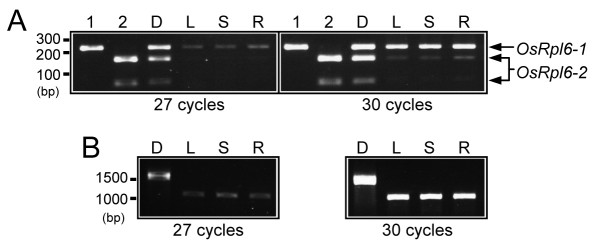
**Semi-quantitative RT-PCR analysis of the rice *rpl6 *genes**. (A) Comparison of the relative amounts of *OsRpl6-1 *and *OsRpl6-2 *cDNAs after 27 (left panel) or 30 cycles of PCR (right panel). Bands derived from each gene are indicated by arrows. Lanes 1 and 2: negative and positive controls for *Dra*I digestion using cloned *OsRpl6-1 *and *OsRpl6-2 *plasmid DNAs as templates, respectively. Note that the *OsRpl6-2 *gene has a *Dra*I site in its coding region (Fig. 1A), resulting in two bands after digestion, whereas the *OsRpl6-1 *gene does not. Lane D: *Dra*I digestion of *OsRpl6-1 *and *OsRpl6-2 *DNA products, which were amplified using rice genomic DNA as a template instead of first-strand cDNAs. Lanes L, S and R: from left to right, relative amounts of *OsRpl6-1 *and *OsRpl6-2 *cDNAs in mature leaf, leaf sheath and root. (B) Amplification of rice *Actin *genes as an internal control. Molecular size standards are shown on the left.

We examined the transcription of the *OsRpl6-1 *and *OsRpl6-2 *genes using reverse transcription-PCR (RT-PCR). Because of difficulty in designing primers specific for each *rpl6 *gene, *OsRpl6-1 *and *OsRpl6-2 *cDNAs were amplified using a common primer pair, P7/P8 (Fig. 1A; Table 1), followed by restriction digestion to distinguish between their products (see Methods). The RT-PCR analysis showed that amplification of *OsRpl6-2 *cDNA was much lower than that of *OsRpl6-1 *cDNA (Fig. 3A, lanes L, S and R). This indicates that the two *rpl6 *genes differ in the amount of transcripts produced.

### The 5'-UTR of OsRpl6-1 is homologous to various rice sequences

**Figure 4:**
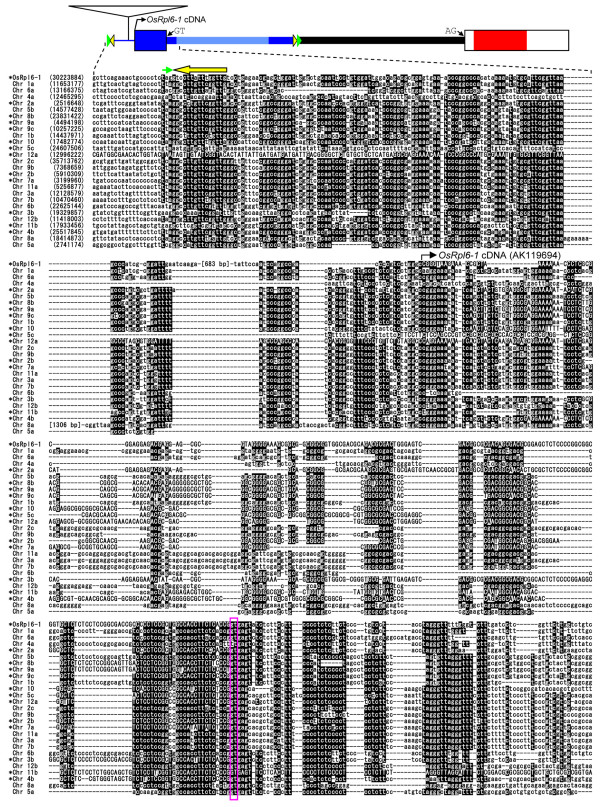
**Alignment of sequences homologous to the region around the 5'-UTR of *OsRpl6-1 *in the rice genome**. See Figure 1 for a schematic representation of *OsRpl6-1 *above the sequence alignment. Sequence names are shown on the left, which are designated according to their chromosome (Chr) numbers except for *OsRpl6-1*. When more than one sequence exists on the same chromosome, they are distinguished as a, b or c, relative to their nucleotide positions in the database. The first nucleotide positions in the alignment are indicated within parentheses, and correspond to those of the Rice Annotation Project Database Build 4 [21]. Nucleotides conserved in >60% of sequences are highlighted. Gaps were introduced to maximize the sequence identity. The presence of the corresponding cDNA in the given direction is indicated by asterisks at the sequence names. Sequences corresponding to the cDNAs are capitalized in the alignment. The first nucleotide of the *OsRpl6-1 *cDNA clone [accession no. AK119694] is indicated by a bent arrow. The GT dinucleotides at the 5'-border of the intron are shown within an empty box with a pink outline. Putative terminal inverted repeats (TIRs) and target site duplications (TSDs) are indicated by thick yellow arrows and small green arrows, respectively, according to the colors in Figure 1B.

**Figure 5:**
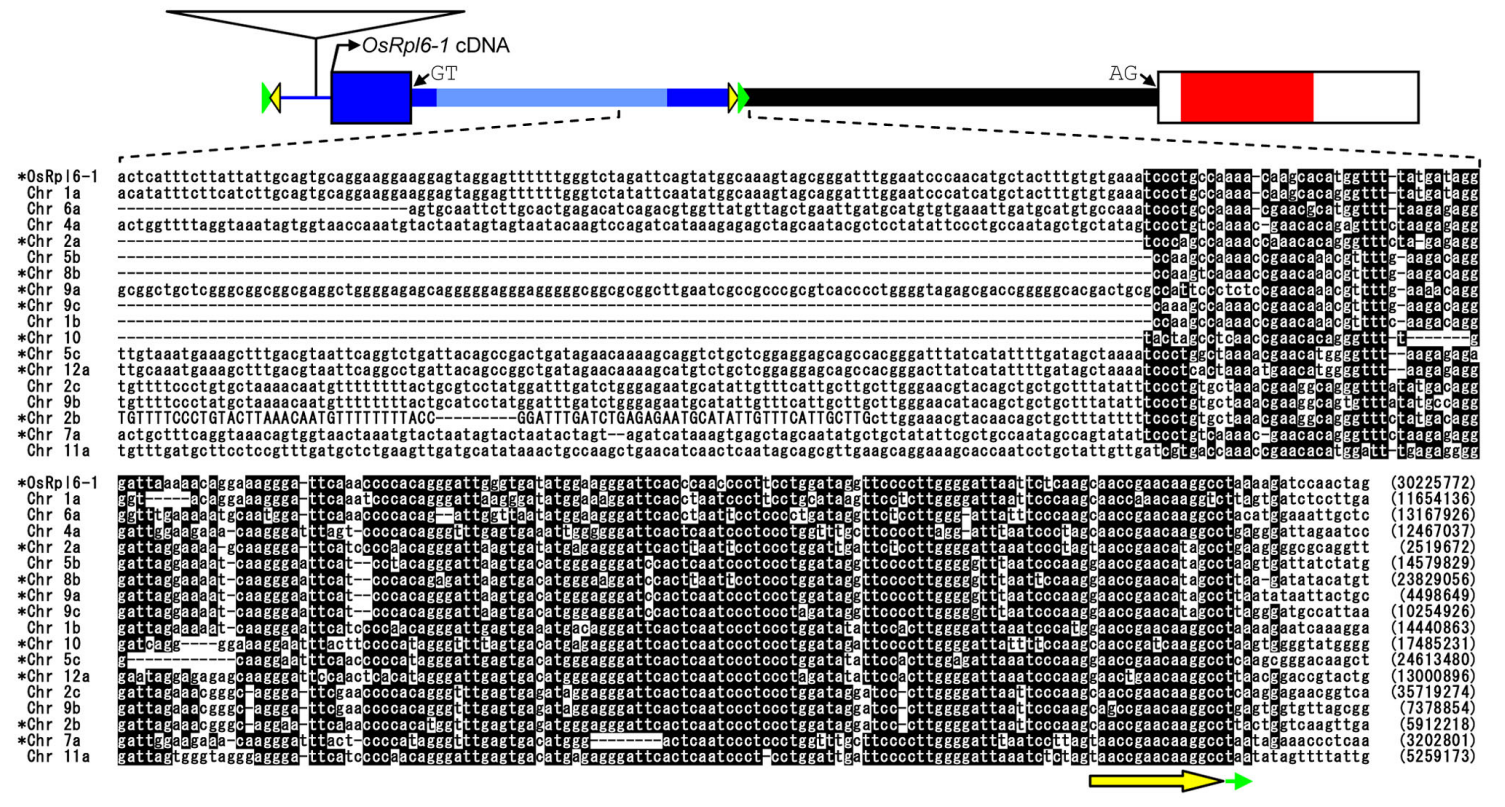
**Alignment of sequences homologous to the central part of the *OsRpl6-1 *intron in the rice genome**. Only sequences containing the last part of the conserved sequence segment are shown. The last nucleotide positions in the alignment are indicated within parentheses. Other captions are similar to those in Figure 4.

In contrast to the similar coding sequences of the two *rpl6 *copies, their 5'-noncoding regions differ near the center of the intron (Fig. 1A). A database search was conducted to determine the origin of these 5'-sequences. There are several sequences homologous the region around the 5'-UTR of *OsRpl6-1 *(Figs. 1B and 4, blue color) in the rice genome. These sequences are distributed on all rice chromosomes (Fig. 4A, designated Chr xx according to their chromosomal positions). Additional homologous sequences may be revealed when divergent and fragmented sequences are taken into account. The homology starts upstream from the 5'-end of *OsRpl6-1 *cDNA (Fig. 4A, bent arrow) and extends into the 5'-part of the intron. The Chrs 4a, 5c and 12a lack about 110, 180 and 60 bp of sequences from the beginning of the conserved region, respectively, probably because of subsequent deletion events. The GT dinucleotide at the 5'-border of the *OsRpl6-1 *intron is also conserved in all the Chr sequences (Fig. 4, empty box with pink outline), except for Chr 4a. Eleven of these sequences (Chrs 2a, 2b, 3b, 4b, 5c, 7a, 8b, 9a, 9c, 10 and 12a) are transcribed and spliced at this site because their corresponding cDNAs are present in the database [GenBank accession nos. AK102901, CI553335, CI643836, CI733280, AK064122, AK107811, CI076773, CI643779, CI241123, CI239335 and AK067455, respectively] (Fig. 4, asterisks in sequence names). In addition, we detected another conserved DNA segment downstream of the above-mentioned homologies in 18 sequences (*OsRpl6-1*, and Chrs 1a, 1b, 2a, 2b, 2c, 4a, 5b, 5c, 6a, 7a, 8b, 9a, 9b, 9c, 10, 11a and 12a) (Fig. 5). In *OsRpl6-1*, this downstream conserved segment is located near the center of the intron, upstream of the sequence homologous in the two *rpl6 *genes (Fig. 1A, shaded region).

### Conserved regions have characteristics of a transposable element (TE)

The upstream (Fig. 4) and downstream (Fig. 5) conserved segments are presently annotated in the database as members of two separate nonautonomous TEs, MERMITE18F and ECSR [24], respectively, but they have not been characterized in detail. Sequence alignment and analysis of the two conserved segments revealed the following. (1) The segments have a common terminal inverted repeat (TIR) composed of a 15-bp consensus sequence, GGCCTTGTTCGGTTG (Figs. 4 and 5, thick yellow arrows). (2) A potential 3-bp direct repeat occurs just outside of the TIR (Figs. 4 and 5, small green arrows). Although not all direct repeats were perfectly conserved between the ends of the sequences, they could represent target-site duplications (TSDs), which are generally caused by TE insertions. (3) A database search using the sequences flanking the conserved segments detected two putative *r*elated-to-*e*mpty-*sites *(RESites). The RESites are sequences that are homologous to TE-bearing sequences but lack the TE insertion, which indicates the past movement of TEs and their TSD sequences [25]. In one instance, the sequence flanking Chr 2b was nearly identical to that of its RESite (Fig. S1A in Additional file 1). In the second instance, high homology was evident between Chr 9c and its RESite, although a few indels were observed (Fig. S1B in Additional file 1). These results strongly suggest that the two conserved segments were moved as a single TE because the insertion of two such segments in such close proximity and in the same direction by two separate events is highly unlikely.

### Classification of TEs associated with the 5'-UTR of OsRpl6-1 as a member of the PIF/Harbinger superfamily

Among the putative TEs re-characterized in this study, proteins predicted from the internal regions of Chrs 2c, 5c and 9b had 78%–88% similarity to a transposase from *Os-PIF1 *(data not shown). The *Os-PIF1 *is a rice homologue of maize *P *instability factor alpha (*PIFa*), an active class II DNA transposon [26]. The *PIF *family has recently been associated with the nonautonomous miniature inverted transposable element (MITE), *Tourist *[27]. In fact, the consensus TIR sequence of the putative TEs observed in our study (GGCCTTGTTCGGTTG) (Figs. 4 and 5) was similar to that of *Tourist*-like MITEs in maize, barley and *Sorghum *[27,28] and *OsPIF *families [29]. These results indicate that the conserved sequence segments associated with the 5'-UTR of *OsRpl6-1 *are a single TE belonging to the *PIF/*Harbinger superfamily. Of these TEs, Chr 2c seems to encode an entire transposase, whereas Chr 5c and 9b may be pseudogenes because of lacking the complete coding region for transposase. The others, including one within *OsRpl6-1*, are probably nonautonomous elements because they did not contain ORFs nor did their predicted proteins have significant homologies to any characterized proteins in the given direction (data not shown).

## Discussion

**Figure 6:**
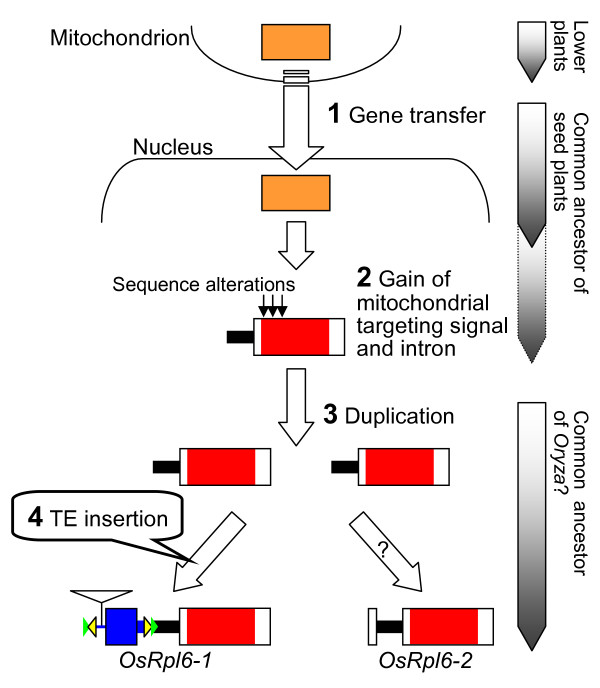
**A proposed model for the gene transfer and activation of rice *rpl6***. The numbers in the figure correspond to those of the subtitles in the Discussion. The mitochondrial *rpl6*-derived coding region is colored with orange. The coding regions of nucleus-encoded *rpl6 *copies with mitochondrial targeting ability are colored with red. The 3'-part of the intron is indicated by a thick black line. Other elements are indicated as in Figure 1. The predicted evolutionary timing of each event is indicated on the right.

Based on the results of this study, we propose a model for the gene transfer and subsequent events of rice *rpl6 *(Fig. 6).

### 1. Gene transfer of rpl6 from the mitochondrion to the nucleus

It has been proposed that a mitochondrially encoded *rpl6 *gene had been transferred to the nucleus prior to the emergence of angiosperms [7,8]. We assume that the transfer already occurred in the common ancestor of seed plants (Fig. 6, step 1) because the *rpl6 *gene is absent from the mitochondrial genome of a gymnosperm, *Cycas taitungensis *[30]. In addition, *rpl6 *cDNAs are found from gymnosperms *Cryptomeria japonica, Cycas rumphii, Pinus pinaster, Pseudotsuga menziesii *and *Zamia vazquezii *in the database [their representative accession nos. are BY896644, CB092074, BX248809, CN638760 and FD772805, respectively], although the presence of *rpl6 *cDNAs does not readily indicate the nuclear localization of genes. This situation differs from the evolution of other ribosomal protein genes, which underwent recent gene transfer events during the course of angiosperm evolution (e.g., *rps10 *gene) [15].

### 2. Gain of a mitochondrial targeting sequence and an intron

Since neither of the proteins predicted from the two rice *rpl6 *genes contained an apparent N-terminal extension for a presequence, the targeting signal seems to have been derived from sequence alterations within the mature N-terminal coding region (Fig. 6, step 2), as with the case of rice *rps10 *[31]. The presence of an embedded targeting signal here was indicated by the results of GFP assays (Fig. 2). During the GFP analysis, small aggregations were occasionally observed (data not shown). We speculate that the efficiency of mitochondrial targeting varies according to cellular or physiological conditions. Incomplete or slow protein targeting has been observed in the sweet potato ATPase δ-subunit with an atypical mitochondrial targeting signal [32]. At any rate, the acquisition of the targeting signal would have occurred prior to the duplication event because both rice RPL6 proteins have similar mitochondrial targeting abilities. This step may predate the common ancestor of seed plants because seed plants RPL6 proteins seem to lack a presequence as described. The 3'-part of an intron would also have been acquired during this step, based on the fact that both of the *OsRpl6-1 *and *OsRpl6-2 *sequences share a similarity in the 3'-terminal region of the intron (Fig. 1, shaded region).

### 3. Duplication of the nuclear rpl6 gene

The *rpl6 *gene would have been duplicated via genomic DNA, resulting in two *rpl6 *copies (*OsRpl6-1 *and *OsRpl6-2*) on different chromosomes (Fig. 6, step 3). Although we did not conduct Southern blot analysis to determine their copy numbers, the presence of the two *rpl6 *copies in the rice nucleus is probable because of the accuracy of rice genome sequence data [20] and similarity to numerous *rpl6 *cDNA sequences in the database (data not shown). The duplication event seems to have occurred after the split of the genus *Oryza *from the other monocots, followed by the occurrence of *japonica *and *indica *subspecies, because cDNA sequences corresponding to *OsRpl6-1 *and *OsRpl6-2 *are also present in the *indica *cultivar [their representative GenBank accession nos. are CX108080 and CT862828, respectively] but not in other monocots (data not shown). This assumption is supported by a maximum likelihood (ML) tree based on the 59 nonredundant *rpl6 *cDNAs from 24 angiosperm genera (data not shown). This ML tree also suggests relatively recent duplications in *Glycine, Hordeum, Ipomoea, Petunia *and *Triticum*. Therefore, it is likely that multiple duplication events occurred during angiosperm evolution.

### 4. Acquisition of the 5'-UTR of OsRpl6-1 via a TE

Despite their coding similarity, transcripts of *OsRpl6-2 *were much less abundant than those of *OsRpl6-1 *(Fig. 3). The difference in the quantity of transcripts produced by the *rpl6 *genes might be caused by differences in promoter regions because their 5'-noncoding regions differ from a point near the center of the intron (Fig. 1A). The most striking findings of this study were that numerous sequences homologous to the region around the 5'-UTR of *OsRpl6-1 *were detected in the rice genome and that they presumably belong to a TE family. One can raise a question why numerous putative introns linked to the TEs are spread in the rice genome. One speculation could be that a TE was selected as part of the intron of a cellular gene because it contained a functional element such as a promoter or an enhancer, followed by amplification in the rice genome via transpositions. Alternatively, a TE might have selfishly been spread in the rice genome after capture of the 5'-part of intron from an unknown gene source. The evolutionary relationships of these TEs are unclear because the presence of a number of indels in the sequence alignment (Figs. 4 and 5) precludes fine phylogenetic analysis. These TEs might have been transposed at a relatively early evolutionary stage because most of their RESites are missing. This assumption does not contradict the notion of ancient transfer of the *rpl6 *gene to the nucleus [7,8]. However, the TE within *OsRpl6-1 *should have been integrated after the duplication event of the rice *rpl6 *gene (Fig. 6, step 4) because the *OsRpl6-2 *and *Arabidopsis rpl6 *genes lack such a sequence. The origin and mode of acquisition of the *OsRpl6-2 *5'-UTR is unknown. There is a *Mutator*-like element (MULE) within the 5'-nontranscribed spacer region of *OsRpl6-1 *(Figs. 1B and 4, open triangle). This element seems to have been acquired posteriorly. Such nested TE insertions are characteristic of many kinds of TEs. We infer that this MULE has a minor effect on the expression of *OsRpl6-1 *because it is not conserved among the contemporary transcribed TEs (Fig. 4, asterisks in sequence names). Alternatively, the MULE might act as an enhancer.

The 5'-UTR and intron within the 5'-UTR are generally thought to contain cis-elements that regulate expression at transcriptional and posttranscriptional levels: the former involves promoter and enhancer activities and the latter confers translational efficiency and mRNA stability [33,34]. The evolutionary origins of noncoding regions (e.g., 5'- and 3'-UTRs, promoters and introns) are mostly unknown, as are those of nucleus-encoded mitochondrial genes. Recently, topoisomerase I-mediated homologous recombination has been proposed as a mechanism by which the 5'-UTR was acquired in rice *rpl27 *[18]. In the present report, we describe a novel mechanism for the acquisition of a 5'-UTR via a TE. TEs sometimes transpose in the vicinity of host genes, generating new coding regions and changing gene expression [35]. Among the TEs, MITEs may be sources of cis-acting regulatory elements because of their specific properties. First, MITEs are much more prevalent than other types of TEs in plant genomes. Second, they preferentially insert into genic regions. Finally, MITEs might contain cis-acting elements. Although most of such putative elements have not been demonstrated experimentally, a MITE family that had provided a poly (A) signal has been reported [36]. In addition, it is noteworthy that insertions of a member of the MITE family, *mPing*, may have caused the up- and down-regulation of adjacent genes in rice [37]. We have not determined which cis-acting element causes differences in the amount of transcripts between the two rice *rpl6 *genes (Fig. 3A). However, many reports have already established that the 5'-UTR and intron within the 5'-UTR have promoter and enhancer activity. As an alternative hypothesis, it is also possible to assume that the rice *rpl6 *gene gained basal transcriptional machinery prior to the gene duplication event (Fig. 6, step 2) because both of the rice *rpl6 *copies are transcribed. In this case, the TE within *OsRpl6-1 *might act as an enhancer.

Despite some functional ambiguity, judging from the lines of evidence presented here, our results constitute a plausible explanation for the origin and acquisition of the 5'-noncoding region. The generality of the acquisition of a 5'-UTR via a TE is unclear because of the paucity of genomic information on *rpl6 *genes in other monocots and because many TEs are often poorly conserved except for TIRs and TSDs. In fact, we examined the structure of the TEs that retain the entire TIR and are transcribed (Chrs 2a, 2b, 7a, 8b, 9a, 9c and 10), but failed to find any analogous case of *OsRpl6-1*. Their transcripts ended within a region between the TIR (data not shown) and no association with any other proximal genes was predicted. Therefore, to our knowledge, the *OsRpl6-1 *is presently the only example. Additional genomic data on other plant species and further systematic searches may reveal analogous cases of other transferred mitochondrial genes.

## Conclusion

We have demonstrated the evolutionary origin and acquisition mechanism of the 5'-UTR of a transferred mitochondrial gene. We conclude that the 5'-UTR of the transferred *rpl6 *gene was acquired via a TE. Since the 5'-UTR and intron within the 5'-UTR generally contain transcriptional and posttranscriptional cis-elements, TEs may have constituted sources of cis-elements for the transferred mitochondrial genes.

## Methods

### Database search and nucleotide sequence analyses

Sequences homologous to the rice *rpl6 *gene were sought using the BLAST algorism in the National Center for Biotechnology Information http://www.ncbi.nlm.nih.gov/ and the RAP-DB Build 4 http://rapdb.dna.affrc.go.jp/[21] databases and a rice *rpl6 *cDNA [GenBank accession no. AU184578] as the initial query. No sequence filtering was set. The intron position of each *rpl6 *gene was determined by comparison between the cDNA sequence [accession nos.: AK119694 and CI260120] and the corresponding genomic sequence [locus tags: Os03g0725000 and Os08g0484301]. RESites were detected using flanking sequences immediately outside of the putative TEs as queries in BLAST searches, as described previously [25].

### Construction and visualization of GFP fusion proteins

Portions of *OsRpl6-1 *and *OsRpl6-2 *coding regions were amplified by PCR using primer pairs P1/P2, P3/P4 and P5/P6 (Fig. 1; Table 1). These primer pairs contained regions encoding amino acid positions 1–52 of *OsRpl6-1*, 1–52 of *OsRpl6-2 *and 54–103 of *OsRpl6-1*, respectively. Each amplified fragment was fused in-frame to 5'-upstream or 3'-downstream of S65TsGFP cDNA (kindly provided by Dr. Y. Niwa; [23]) as described previously [31]. The transient expression of GFP fusion proteins in tobacco BY-2 cells was visualized as described previously [38] after staining with a mitochondria-specific dye, MitoTracker Red (Invitrogen, Carlsbad, CA, USA).

**Table 1:** Primers used in this study.

Primer	Sequence
P1	5'-CCTCTTCTCTTGTTGccATGGAG-3'
P2	5'-CAGTACAGCAAATTAccaTGGGTTTG-3'
P3	5'-TTTCCCTGTCTTATTGCcATGGAA-3'
P4	5'-CCAGTACAGCATATTAccaTGGGTTTA-3'
P5	5'-CTGCTTCAAACtgtACaTAATTTGC-3'
P6	5'-GAAAGTAAAATgcGGccGCTCATCC-3'
P7	5'-TTGTTTCTGAAACTTGGTTACAG-3'
P8	5'-TTACTTCTTTTGCTTCTTCCCTGGCTT-3'
P9	5'-AATGG(A/C)AC(C/T)G(A/G)(A/T)ATGGTCAAG-3'
P10	5'-TTAGAAGCA(C/T)TTC(A/C)TGTG(C/G)AC-3'

Underline: restriction sites for *Bsr*GI, *Nco*I and *Not*I.

Lowercase letters: deviations from the original *OsRpl6-1 *sequence to enable introduction of restriction sites.

The locations of primers P1-P8 are shown in Figure 1A.

Primers P9 and P10 were designed from rice *Actin *sequences [39] as described previously [40] with slight modifications.

### RT-PCR analysis

Total RNA was isolated from mature leaves, leaf sheaths and roots of three-month-old rice plants (*Oryza sativa *subsp. *japonica *cv. Nipponbare) using an RNeasy Plant Mini Kit (Qiagen, Valencia, CA, USA). One microgram of total RNA was treated with RNase-free DNase I (Roche Diagnostics, Basel, Switzerland). First-strand cDNAs were synthesized using oligo (dT)_18 _primers and the Advantage RT-for-PCR Kit (Takara Bio, Otsu, Japan). It was difficult to design primers specific for each *OsRpl6 *gene because of a GC-rich sequence in the 5'-region and an interspersed repeat sequence in the 3'-UTR (data not shown). Therefore, *OsRpl6-1 *and *OsRpl6-2 *cDNAs were amplified using a common primer pair, P7/P8 (Fig. 1; Table 1). After 27 and 30 cycles of PCR reaction, the products of each gene were digested with *Dra*I to distinguish between them (see legend for Fig. 3). Rice *Actin *genes [39] were used as an internal control.

## Authors' contributions

NK designed this study, performed database searches and RT-PCR analyses, and drafted the manuscript. MH helped with data interpretation. MF and SA carried out GFP transient assays. MF, SA and NT interpreted the GFP data. All authors read and approved the final manuscript.

## Supplementary Material

Additional file 1 **Figure S1**. **Comparison of sequences with putative transposable elements (TEs) and the homologous sequences of *r*elated-to-*e*mpty-*sites *(RESites)**. (A) Chr 2b and its RESite. (B) Chr 9c and its RESite. The top line and the other lines show transposable elements (TEs) and RESites, respectively. The positions of the first and last nucleotides in the alignment are denoted within parentheses, which correspond to those of the Rice Annotation Project Database Build 4 [21]. Gaps were introduced to maximize the sequence identity. Insertions of TEs in Chrs 2b and 9c are indicated by blue boxes, in which yellow triangles on the right and left borders represent terminal inverted repeats (TIRs). The sizes of insertions are shown above the blue boxes. The predicted target site duplications (TSDs) are colored with green, as in Figures 1, 4 and 5. Click here for file
