# Supplementary material for: Transfer of rice mitochondrial ribosomal protein L6 gene to the nucleus: acquisition of the 5'-untranslated region via a transposable element
Source: BMC Evol Biol. 2008 Nov 14;8:314. doi: 10.1186/1471-2148-8-314 (PMC2631504; doi:10.1186/1471-2148-8-314)
Supplement: Additional file 1 — Figure S1. Comparison of sequences with putative transposable elements (TEs) and the homologous sequences of related-to-empty-sites (RESites). (A) Chr 2b and its RESite. (B) Chr 9c and its RESite. The top line and the other lines show transposable elements (TEs) and RESites, respectively. The positions of the first and last nucleotides in the alignment are denoted within parentheses, which correspond to those of the Rice Annotation Project Database Build 4 [21]. Gaps were introduced to maximize the sequence identity. Insertions of TEs in Chrs 2b and 9c are indicated by blue boxes, in which yellow triangles on the right and left borders represent terminal inverted repeats (TIRs). The sizes of insertions are shown above the blue boxes. The predicted target site duplications (TSDs) are colored with green, as in Figures 1, 4 and 5. [file 1471-2148-8-314-S1.pdf]

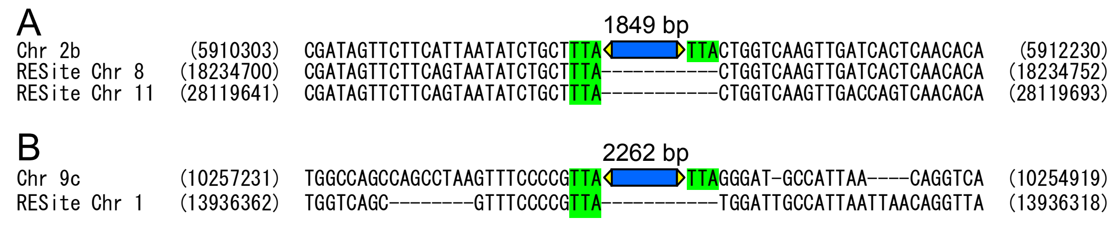
A
1849 bp
Chr 2b (5910303) CGATAGTTCTTCATTAATATCTGCTTTA TTACTGGTCAAGTTGATCACTCAACACA (5912230)
RESite Chr 8 (18234700) CGATAGTTCTTCAGTAATATCTGCTTTA-----------CTGGTCAAGTTGATCACTCAACACA (18234752)
RESite Chr 11 (28119641) CGATAGTTCTTCAGTAATATCTGCTTTA-----------CTGGTCAAGTTGACCAGTCAACACA (28119693)
B
2262 bp
Chr 9c (10257231) TGGCCAGCCAGCCTAAGTTTCCCCGTTA TTAGGGAT-GCCATTAA----CAGGTCA (10254919)
RESite Chr 1 (13936362) TGGTCAGC--------GTTTCCCCGTTA-----------TGGATTGCCATTAATTAACAGGTTA (13936318)
